# Hantavirus-induced disruption of the endothelial barrier: neutrophils are on the payroll

**DOI:** 10.3389/fmicb.2015.00222

**Published:** 2015-03-25

**Authors:** Günther Schönrich, Detlev H. Krüger, Martin J. Raftery

**Affiliations:** Institute of Medical Virology, Helmut-Ruska-Haus, Charité–Universitätsmedizin Berlin, Berlin, Germany

**Keywords:** viral hemorrhagic fever, hantaviruses, immunopathogenesis, neutrophils, neutrophil extracellular traps, vascular hyperpermeability

## Abstract

Viral hemorrhagic fever caused by hantaviruses is an emerging infectious disease for which suitable treatments are not available. In order to improve this situation a better understanding of hantaviral pathogenesis is urgently required. Hantaviruses infect endothelial cell layers *in vitro* without causing any cytopathogenic effect and without increasing permeability. This implies that the mechanisms underlying vascular hyperpermeability in hantavirus-associated disease are more complex and that immune mechanisms play an important role. In this review we highlight the latest developments in hantavirus-induced immunopathogenesis. A possible contribution of neutrophils has been neglected so far. For this reason, we place special emphasis on the pathogenic role of neutrophils in disrupting the endothelial barrier.

## Introduction

Viral hemorrhagic fever (VHF) is caused by viruses belonging to different virus families, one of which is the Bunyaviridae (Schmaljohn and Nichol, 2007). Structurally, hantaviruses have an envelope derived from the host cell membrane. Their genome consists of three negative-strand RNA segments encoding a nucleoprotein (N), two glycoproteins (Gn and Gc), and a RNA-dependent RNA polymerase (Schmaljohn and Nichol, 2007). According to the geographic location of the natural reservoir hosts and the disease syndrome induced, hantaviruses are divided into Old World and New World hantavirus species.

Humans become infected with hantaviruses after inhalation of aerosols derived from excreta of persistently infected but asymptomatic natural reservoir hosts, in general rodents. Depending on the hantavirus species involved the severity of hantavirus-induced disease varies with case fatality rates from less than 1% to up to more than 40% (Jonsson et al., 2010; Krüger et al., 2015). Old World hantavirus species such as Hantaan virus (HTNV) are associated with hemorrhagic fever with renal syndrome (HFRS). After an incubation period of approximately 3 weeks HFRS starts with a febrile phase and further unspecific symptoms. Subsequently, hypotension and oliguria is observed that may finally result in fatal shock. Patients recover after a polyuric phase that starts in the second week of illness. A mild form of HFRS, also termed nephropathia epidemica, with a case fatality rate of less than 1% is endemic in Europe and is in large part due to infection with Puumala virus (PUUV; Mustonen et al., 2013). In contrast, infection with New World hantavirus species such as Sin Nombre virus (SNV) can result in hantavirus cardio-pulmonary syndrome (HCPS; Nichol et al., 1993). In the course of HCPS patients develop pulmonary edema and cardiac failure whereas in HFRS kidney failure is the prominent clinical feature. Andes virus (ANDV) is the most lethal New World hantavirus species with case fatality rates of up to more than 40%. It is the only hantavirus species for which human-to-human-transmission has been reported (Martinez-Valdebenito et al., 2014). Some hantavirus species such as Prospect Hill virus (PHV) are non-pathogenic whereas others such as Tula virus (TULV) cause only sporadically disease (Klempa et al., 2003; Zelena et al., 2013). In China 20,000 to 50,000 HFRS cases are reported annually, which represents 90% of HFRS cases worldwide (Fang et al., 2015).

It is now increasingly apparent that the paradigm of two distinct syndromes induced by Old Word and New World hantaviruses needs to be reconsidered (Krautkrämer et al., 2013; Clement et al., 2014). For example, cardiopulmonary dysfunction can dominate the clinical picture after infection with Old World hantavirus species (Clement et al., 1994; Rasmuson et al., 2011a,b; Gizzi et al., 2013). *Vice versa*, kidney function is also impaired in patients suffering from infection with New World hantavirus species (Pergam et al., 2009; MacNeil et al., 2011).

As with other VHF dysregulation of the endothelial cell (EC) barrier resulting in capillary leakage is the key finding in hantavirus-induced disease (Duchin et al., 1994). The extent of vascular dysfunction determines the severity of the clinical course. So far no preventive or therapeutic strategies for hantavirus-induced disease have been approved by the Food and Drug Administration (Schmaljohn, 2009; Krüger et al., 2011). However, an experimental HCPS DNA vaccine has been successfully tested in non-human primates (Kwilas et al., 2014). Moreover, the HCPS DNA vaccine elicits production of neutralizing human IgG (immunoglobulin G) in *trans*-chromosomal bovines which could be used for passive immunoprophylaxis in humans (Hooper et al., 2014). In this review we will focus on concepts explaining how hantavirus-induced immune responses interfere with the endothelial barrier function and briefly mention also non-immunological mechanisms.

## Non-Immunological Mechanisms

Hantaviruses infect and replicate in EC cultures without causing any cytopathic effect or increasing permeability (Pensiero et al., 1992; Temonen et al., 1993; Khaiboullina et al., 2000; Sundstrom et al., 2001). However, in the presence of vascular endothelial growth factor (VEGF) replication of HTNV or ANDV in human umbilical EC downregulates vascular endothelial (VE)-cadherin, a major component of adherens junctions, thereby disrupting the endothelial barrier (Gavrilovskaya et al., 2008; Gorbunova et al., 2010; Li et al., 2012). Recently, VE-cadherin degradation was observed even in the absence of exogenous VEGF after ANDV infection of primary human pulmonary microvascular EC (Shrivastava-Ranjan et al., 2010). This was not confirmed in another experimental setup using *in vitro* capillary blood vessels (Taylor et al., 2013). In this system it was found that infection with HTNV or ANDV results in activation of the kallikrein–kinin system and liberation of bradykinin, a potent inducer of vascular permeability (Taylor et al., 2013). In accordance, a bradykinin receptor antagonist improved the clinical outcome in a case of PUUV infection (Antonen et al., 2013). Finally, glomerular EC infected with PUUV show disruption of cell-to-cell contacts (Krautkrämer et al., 2011).

## Hantaviral Immunopathogenesis

Both innate and adaptive as well as humoral and cellular immune mechanisms contribute to hantavirus-associated disease. Human dendritic cells (DC) are highly mobile and bridge innate and adaptive immunity. DC reside at the pathogen-host interface in peripheral tissue including the respiratory mucosa and alveoli of the lung. They can push their dendritic projections into the airway lumen thereby “snorkeling” through the epithelial-tight junctions (Jahnsen et al., 2006). Thus, DC may become infected with hantavirus in the lung shortly after inhalation of viral particles. In accordance, human DC are susceptible to infection with HTNV and ANDV *in vitro* (Raftery et al., 2002; Marsac et al., 2011). Moreover, monocytes infected with HTNV develop into DC-like cells (Markotic et al., 2007; Schönrich et al., 2008). DC might act as a Trojan horse helping the pathogens to disseminate within the human organism and finally infect EC in various organs. Alternatively, DC may become infected later when they get in contact with the already infected human EC barrier. In striking contrast to most other DC-tropic viruses both Old World and New World hantavirus species induce DC maturation *in vitro* (Raftery et al., 2002; Marsac et al., 2011). This implies that in humans hantavirus-infected DC migrate to the draining lymph nodes and induce a vigorous adaptive immune response.

In accordance, histopathological analysis of tissue collected from fatal human HCPS cases has revealed strong mononuclear cell infiltrates especially in lung tissue (Nolte et al., 1995; Zaki et al., 1995). Similarly, endobronchial mucosal biopsies and bronchoalveolar lavage fluid from HFRS patients revealed activated CD8^+^ T cells and strong upregulation of vascular cell adhesion molecule 1 (VCAM-1) at the site of infection (Rasmuson et al., 2011b). Animal models of HCPS based on non-human primates and Syrian hamsters confirmed that an excessive and aberrant tissue-specific host response correlates with increased vascular hyperpermeability (Safronetz et al., 2015). For unknown reasons, however, T cell depletion neither influenced the viral load nor the clinical course of HCPS in Syrian hamsters. Intriguingly, most of the host genes that are linked to hantavirus disease severity are associated with abnormal immune responses or even autoimmune diseases (Charbonnel et al., 2014). In line with this view elevated levels of autoantibodies to nuclear antigen are found in hantavirus-infected patients (Raftery et al., 2014).

## Activation of Endothelial Cells

Immunohistological studies of kidney biopsies derived from HFRS patients revealed that EC become activated during PUUV infection and increase expression of chemokines and adhesion molecules such as intercellular adhesion molecule 1 (ICAM-1), E-Selectin, and VCAM-1 (Temonen et al., 1996). The latter are important for regulating the interaction of EC with immune cells (Razakandrainibe et al., 2013). It is questionable whether hantavirus directly upregulate adhesion molecules on EC (Sundstrom et al., 2001; Geimonen et al., 2002; Yu et al., 2014). It has been established, however, that immune cells stimulated during hantavirus infection release tumor necrosis factor alpha (TNF-α), a strong inducer of adhesion molecules in EC (Pober, 2002). The chemokines that are upregulated during hantavirus infection include interleukin (IL)-8 (Klingstrom et al., 2008; Sadeghi et al., 2011; Libraty et al., 2012; Kyriakidis and Papa, 2013), a key neutrophil-recruiting chemokine and activator (Amulic et al., 2012). Intriguingly, in some studies IL-8 levels were positively correlated with severe acute disease suggesting that it is part of an important pathogenic link (Libraty et al., 2012; Kyriakidis and Papa, 2013). Moreover, expression of HLA (human leucocyte antigen) class I molecules is increased on EC (Kraus et al., 2004). These include HLA-E (Bjorkstrom et al., 2011) which serves as a ligand for the activating NK (natural killer) cell receptor NKG2C. Thus, hantavirus-infected EC can interact with a variety of immune effector cells such as HLA class I-restricted CD8^+^ T cells, HLA-E stimulated NK cells and neutrophils.

## Cytotoxic Immune Cells

Cytotoxic activity of activated immune cells may eliminate hantavirus-infected EC thereby causing vascular leakage. A SNV-specific CD8^+^ T cell line lysed HLA-matched SNV-infected EC thereby increasing vascular permeability (Hayasaka et al., 2007). Moreover, involvement of T cells is also supported by genetic susceptibility studies (Terajima and Ennis, 2011). In accordance, researchers have recently detected enhanced endothelial repair activity in HFRS patients (Krautkrämer et al., 2014). A role for cytotoxic immune mechanisms is further supported by increased serum levels of perforin and granzyme B (Klingstrom et al., 2006) as well as cell-free DNA (Outinen et al., 2012a; Raftery et al., 2014) in HFRS patients. However, histopathological examination of tissue from fatal HCPS cases did not reveal necrosis or any overtly visible lesions that can account for the vascular leakage in HCPS patients (Lukes, 1954; Nolte et al., 1995; Zaki et al., 1995). This may be due to difficulties in visualizing small but functionally relevant morphological correlates of endothelial damage. Moreover, it is possible that apoptotic EC are immediately phagocytosed by macrophages or neutrophils.

There is evidence that hantavirus-infected EC are protected, at least to some degree, from attack by cytotoxic T cells and NK cells (Gupta et al., 2013). However, uninfected EC are susceptible to cytotoxic attack and might be prone to bystander killing. For example, a subset of NK cells is activated through increased HLA-E expression on hantavirus-infected EC and may subsequently attack uninfected EC (Braun et al., 2014). This bystander NK attack could be facilitated by the fact that uninfected cells express less inhibitory HLA class I molecules on the cell surface than hantavirus-infected EC (Kraus et al., 2004; Lalwani et al., 2013).

## Neutrophils

Metchnikoff (1887) first postulated that polymorphonuclear cells release substances that damage EC function. Nevertheless, neutrophils have been overlooked in models of hantavirus-induced disease although they represent the most abundant type of immune cell. In fact, neutrophil-rich infiltrates were reported in HCPS patients (Zaki et al., 1995). Moreover, increased numbers of neutrophils with band cell morphology are observed in the blood during hantavirus-associated disease (Hjelle et al., 1995; Zaki et al., 1995). This neutrophil subtype represents most likely a typical left-shift response that is usually found after bacterial challenge and regulates T cell responses (Pillay et al., 2012; Nauseef and Borregaard, 2014). Recent research indicates that neutrophils can contribute generally to hantavirus-induced immunopathogenesis. Upon interaction with activated EC neutrophils undergo NETosis (Gupta et al., 2010; Saffarzadeh et al., 2012), a recently discovered form of programmed neutrophil cell death (Brinkmann et al., 2004). It is characterized by the generation and release of neutrophil extracellular traps (NETs). NETs are a fibrillary network composed of a double-stranded DNA backbone and coated with histones as well as granule molecules such as myeloperoxidase, elastase and cathepsin G. NETosis in close proximity to EC is harmful and results in increased vascular permeability (Gupta et al., 2010; Villanueva et al., 2011; Saffarzadeh et al., 2012).

**FIGURE 1:**
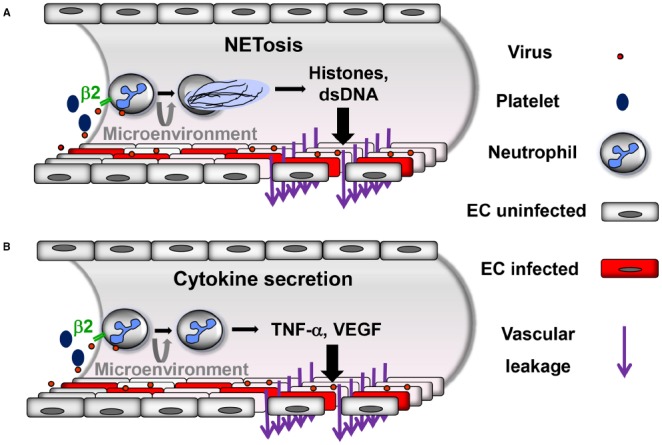
**Proposed neutrophil-mediated mechanisms contributing to vascular leakage during hantavirus infection.** Neutrophils are activated through virus-induced β2 integrin signaling. Activated platelets also stimulate neutrophils through β2 integrins. Depending on the β2 integrin ligands involved and possibly further microenvironmental stimuli neutrophils can be activated in a different way resulting in **(A)** NETosis or **(B)** secretion of inflammatory cytokines such as TNF-α or VEGF. In both cases increased vascular leakage is generated, although most likely by distinct mechanisms.

Neutrophils express β2 integrins, i.e., β2αL (CD18/CD11a), β2αM (CD18/CD11b) and β2αX (CD18/CD11c; Langereis, 2013). A recent study has demonstrated that hantaviruses strongly activate neutrophils through β2 integrin signaling resulting in NETosis (Raftery et al., 2014). In addition, activated platelets recruit neutrophils rapidly to the site of inflamed EC during VHF. Subsequently, platelet-leukocyte aggregation is mediated by the interaction of platelet proteins with β2 integrins on neutrophils (Zapata et al., 2014). Several infection models have demonstrated that platelet-neutrophil interactions through β2 integrins result also in NETosis (Clark et al., 2007; Caudrillier et al., 2012; McDonald et al., 2012; Jenne et al., 2013). Thus, β2 integrins may act as a master switch of NETosis during VHF (Figure 1A).

In accordance with hantavirus-induced NETosis, high levels of extracellular histones are found in sera from hantavirus-infected patients (Raftery et al., 2014; Vaheri et al., 2014). Histones are known to cause microvascular injury and mediate death in sepsis (Xu et al., 2009). Moreover, thrombocytopenia, prolonged prothrombin time and fibrin deposition are hallmarks of hantavirus-induced disease (Laine et al., 2010) and are observed upon histone injection into mice (Fuchs et al., 2011). In fact, extracellular nucleosomes derived from neutrophils induce formation of thrombosis in microvessels which is regarded as an innate host defense mechanism (Massberg et al., 2010). Importantly, depletion of neutrophils prevents pneumonia and vascular hyperpermeability in the SCID (severe combined immunodeficiency) mouse model of hantavirus infection (Koma et al., 2014). The observation that methylprednisolone treatment is not beneficial for HCPS patients is also in accordance with hantavirus-induced NETosis playing an important pathogenic role (Vial et al., 2013) as corticosteroids do not suppress NET formation (Lapponi et al., 2013).

Neutrophils may also contribute to microvascular plasma protein leakage by mechanisms other than NETosis (Figure 1B). Depending on the β2 integrin ligand involved in signaling and further as yet unknown microenvironmental stimuli neutrophils may be activated without undergoing NETosis. After adhering to activated endothelium and crawling along EC neutrophils start to transmigrate and release TNF-α which strongly increases vascular permeability (Finsterbusch et al., 2014). Subsequent binding of TNF-α to its receptor on EC induces endocytosis and degradation of VE-cadherin (Schulte et al., 2011). Similarly, stimulated neutrophils also secrete VEGF (Taichman et al., 1997), an important mediator of VE-cadherin degradation in hantavirus-infected EC (Gavrilovskaya et al., 2008; Gorbunova et al., 2010; Shrivastava-Ranjan et al., 2010; Li et al., 2012). In accordance, high VEGF serum levels are found during HFRS and HCPS (Shrivastava-Ranjan et al., 2010; Gavrilovskaya et al., 2012; Ma et al., 2012). Taken together, neutrophils represent a long-sought missing piece in the puzzle of hantaviral immunopathogenesis.

## Complement System

There is compelling evidence that the severity of HFRS symptoms correlates with the degree of complement activation (Paakkala et al., 2000; Sane et al., 2012). The complement system functions as an important inducer of vascular leakage alongside the kinin and the coagulation system (Bossi et al., 2011). During acute HFRS complement is activated by pentraxin-related protein 3 (PTX3), which represents a humoral pattern recognition receptor (Outinen et al., 2012b). Intriguingly, PTX3 is stored in neutrophil granules and released upon outside-in signals through integrins (Jaillon et al., 2007; Razvina et al., 2014). The soluble complement components C3a and C5a generated during complement activation by antibodies and PTX3 not only induce cytoskeletal rearrangements in EC but also IL-8 secretion (Monsinjon et al., 2003). Consequently, PTX3 attracts more neutrophils to the endothelial barrier aggravating vascular inflammation.

## Inflammatory Cytokines

High levels of proinflammatory cytokines are detected in sera from hantavirus-infected patients especially TNF-α (Linderholm et al., 1996; Mori et al., 1999; Borges et al., 2008; Klingstrom et al., 2008; Sadeghi et al., 2011; Saksida et al., 2011; Libraty et al., 2012; Kyriakidis and Papa, 2013). TNF-α is released by activated antiviral immune cells such as neutrophils, NK cells and CD8^+^ T cells as well as hantavirus-infected DC and macrophages (Raftery et al., 2002; Marsac et al., 2011; Shin et al., 2012).

TNF-α represents a double-edged sword. On one side it may help to control hantaviral dissemination by purging virus from infected cells through non-cytolytic mechanisms (Khaiboullina et al., 2000; Guidotti and Chisari, 2001). On the other side, if it is administered exogenously in quantities that are found during hantavirus infection, vascular leakage and respiratory distress are induced (Tracey and Cerami, 1994; Wimer, 1998). Local release of TNF-α at the EC interface could increase vascular permeability by direct and indirect mechanisms. Firstly, TNF-α not only upregulates adhesion molecules such as ICAM-1, a natural ligand for β2 integrin, but also IL-8. This cytokine both recruits and activates neutrophils, and furthermore induces NETs (Brinkmann et al., 2004). Secondly, TNF-α can directly increase vascular permeability by inducing cytoskeletal rearrangements resulting in redistribution of human microvascular endothelial tight junctions (Blum et al., 1997; Ozaki et al., 1999). Hantaviruses may further enhance this direct TNF-α effect as HTNV-infected EC show prolonged hyperpermeability after exposure to TNF-α in comparison to uninfected control cells (Niikura et al., 2004). The pivotal role of TNF-α in hantaviral immunopathogenesis may explain the relatively poor activity of ribavirin in HCPS; it blocks ANDV replication and suppresses release of some inflammatory mediators but not TNF-α (Khaiboullina et al., 2013).

A high-producing TNF-α genotype (polymorphism at position –308) was linked to more severe HFRS in Finish patients although not independently of the HLA-B8-DR3 haplotype (Kanerva et al., 1998; Makela et al., 2002). This high-producing TNF-α genotype was also more frequently found in HCPS patients than in seropositive individuals without HCPS (Borges et al., 2010). Another study in Belgium showed a link between a low-producing TNF-α genotype (polymorphism at position –238) with more severe HFRS (Maes et al., 2006). This discrepancy may be reconciled by assuming that TNF-α release at the hantavirus-infected EC barrier must be tightly controlled. If there is not enough TNF-α the virus may replicate and disseminate more vigorously especially as hantavirus N protein can interfere with signaling through the TNF receptor (Taylor et al., 2009; Ontiveros et al., 2010). This likely increases vascular permeability due to non-immunological effects of viral particles on subcellular structures. On the other hand, too much local TNF-α allows better control of the virus but at the same time may increase immune-mediated damage.

## Concluding Remarks

**FIGURE 2:**
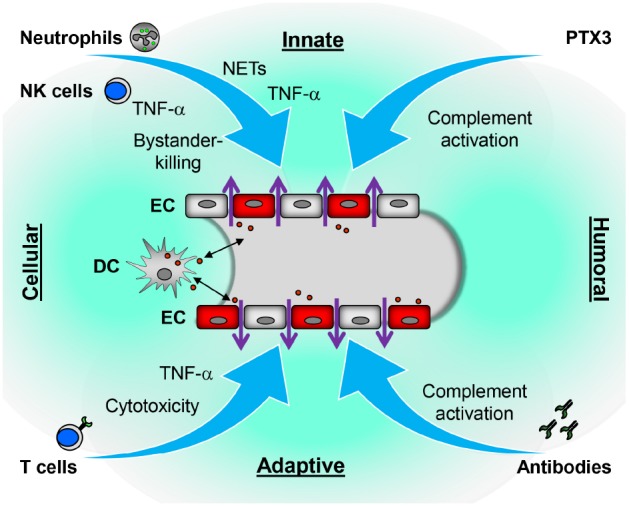
**Proposed immune mechanisms contributing to hantavirus-induced disruption of the endothelial barrier.** Both cellular and humoral components of innate and adaptive immune responses could contribute to vascular leakage of hantavirus-infected vessels. In response to hantavirus-infected EC, neutrophils generate NETs or may secrete inflammatory cytokines such as TNF-α which directly or indirectly increase vascular permeability. The humoral pattern recognition receptor PTX3 and antibodies activate complement. Activated complement components induce cytoskeletal rearrangements in EC further increasing dysfunction of the EC barrier. NK cells may kill bystander EC or also secrete TNF-α. DC may carry the virus from lung tissue to EC of the microvasculature in various organs or become infected after interaction with virus-infected EC. As hantavirus-infected DC mature they migrate to draining lymph nodes to initiate a vigorous CD8^+^ T cell response. The latter could contribute to vascular leakage by direct killing of hantavirus-infected EC or, more likely, by releasing TNF-α.

Humoral as well as cellular mechanisms of the adaptive and innate immune system contribute to hantavirus-induced disruption of the endothelial barrier (Figure 2). Intriguingly, neutrophils which so far have not been regarded as a player in hantavirus-induced immunopathogenesis seem to be important. NETs as well as neutrophil-derived factors such as VEGF, PTX3, and TNF-α can cause vascular dysfunction. Further studies are needed to reveal whether strategies aiming at neutrophil function can prevent hantavirus-induced immunopathogenesis. Furthermore, it is possible that NETs and other neutrophil-derived mediators of vascular hyperpermeability play a role in VHF caused by members of other virus families.

### Conflict of Interest Statement

The authors declare that the research was conducted in the absence of any commercial or financial relationships that could be construed as a potential conflict of interest.
